# The Innate Immune System in Alzheimer's Disease

**DOI:** 10.1155/2013/576383

**Published:** 2013-10-02

**Authors:** Allal Boutajangout, Thomas Wisniewski

**Affiliations:** ^1^Department of Neurology, New York University School of Medicine, Alexandria East River Science Park, 450 East 29th Street, Room 802, New York City, NY 10016, USA; ^2^Psychiatry Department, New York University School of Medicine, Alexandria East River Science Park, 450 East 29th Street, Room 802, New York City, NY 10016, USA; ^3^Physiology and Neuroscience Department, New York University School of Medicine, Alexandria East River Science Park, 450 East 29th Street, Room 802, New York City, NY 10016, USA; ^4^King Abdulaziz University, School of Medicine, Jeddah, KAU 21589, Saudi Arabia; ^5^Pathology, New York University School of Medicine, Alexandria East River Science Park, 450 East 29th Street, Room 802, New York City, NY 10016, USA

## Abstract

Alzheimer's disease (AD) is the leading cause for dementia in the world. It is characterized by two biochemically distinct types of protein aggregates: amyloid **β** (A**β**) peptide in the forms of parenchymal amyloid plaques and congophilic amyloid angiopathy (CAA) and aggregated tau protein in the form of intraneuronal neurofibrillary tangles (NFT). Several risk factors have been discovered that are associated with AD. The most well-known genetic risk factor for late-onset AD is apolipoprotein E4 (ApoE4) (Potter and Wisniewski (2012), and Verghese et al. (2011)). Recently, it has been reported by two groups independently that a rare functional variant (R47H) of TREM2 is associated with the late-onset risk of AD. TREM2 is expressed on myeloid cells including microglia, macrophages, and dendritic cells, as well as osteoclasts. Microglia are a major part of the innate immune system in the CNS and are also involved in stimulating adaptive immunity. Microglia express several Toll-like receptors (TLRs) and are the resident macrophages of the central nervous system (CNS). In this review, we will focus on the recent advances regarding the role of TREM2, as well as the effects of TLRs 4 and 9 on AD.

## 1. Introduction

Alzheimer's disease is the most common cause of dementia globally [1]. AD is characterized by the presence of amyloid *β* (A*β*) deposits in the forms of parenchymal amyloid plaques and congophilic amyloid angiopathy (CAA) as well as aggregated tau protein in the form of neurofibrillary tangles (NFT). These lesions are associated with neuronal loss and synaptic damage, which produce the cognitive dysfunction which characterizes AD. Mutations in three genes have been shown to cause early-onset AD (EOAD): the amyloid precursor protein (APP), Presenilin 1 (PS1), and Presenilin 2 (PS2) [2, 3]. Mutations associated with these genes affect <1% of all AD patients and are not informative regarding the causes of the much more common late-onset AD (LOAD) [2, 3]. Inheritance of the apolipoprotein E (ApoE4) allele is the major genetic risk factor that is associated with late onset AD [4, 5]. Several environmental factors are known as risk factors for LOAD including, aging, head trauma, type 2 diabetes, hypertension, hypercholesterolemia, and vascular pathology [6].

Recently, two independent groups of investigators have identified a rare variant in the gene encoding the triggering receptor expressed on myeloid cells 2 protein (*TREM2*), which is predicted to result in a R47H substitution that causes an ~3-fold increase in the susceptibility to LOAD. Although the odds ratio of Trem2 R47H is comparable to the presence of a single copy of ApoE4, this variant has a population frequency of only ~0.3% [7–9]. TREM2 is located on chromosome 6p21.1 and encodes five exons. It is a transmembrane glycoprotein, made up of an extracellular immunoglobulin-like domain, a transmembrane domain, and a cytoplasmic tail, that couples with DAP12 for its signaling function [10]. TREM2 is expressed in microglia, macrophages, osteoclasts, and dendritic cells. TREM2 was initially identified as a phagocytic receptor of bacteria [11]. It recognizes anionic lipopolysaccharides (LPS) on the surface of bacteria, via signaling through DAP12, triggering their phagocytosis. Other pattern recognition receptors which have been shown to play a critical part in macrophage/microglial function and have a role in AD-related pathology are the Toll-like receptors (TLRs) [12–14]. In this review, we will focus on the potential roles of TLRs and TREM2 in AD.

## 2. TREM2 in AD

The *TREM2* gene encodes 5 exons that code for a 693 pb DNA which translated into 230 amino-acids called TREM2 [15–17]. In the normal brain, TREM2 is highly expressed in white matter, hippocampus, and neocortex, while low levels are found in the cerebellum of the human brain [18]. This distribution of TREM2 expression is consistent with the distribution of AD-related pathology. In AD animal models, it has been reported that TREM2 is upregulated in amyloid plaque-associated microglia, such as in aged APP23 mice [19], a result which was confirmed by another group [20]. In AD model TgCRND8 mice TREM2 mRNA was increased, correlating with the rise in A*β* levels [7].

 TREM2 is a phagocytic receptor of bacteria [11] and forms a receptor signaling complex with the TYRO protein tyrosine kinase binding protein (TYROBP, also called DAP12), that triggers phagocytosis and the release of reactive oxygen species [21]. TREM-2 is defined as an innate immune receptor expressed on the cell surface of microglia, macrophages, osteoclasts, and immature dendritic cells [22]. Microglia play a key role in the immune response in the central nervous system (CNS) and are the resident innate immune cells responsible for the early control of infections. TREM2 is known to have anti-inflammatory properties; it suppresses inflammatory responses by repression of cytokine production and secretion [23]. TREM2 reduces macrophage activation and inhibits cytokine production in response to both TLR2 and TLR4 ligands zymosan and LPS [24, 25]. Conversely reduction of *TREM2* expression by either RNA interference or by targeted gene deletion amplified inflammatory cytokine responses by macrophages following stimulation of multiple different TLRs including TLR2, 4, and 9 [26]. Hence, it has been speculated that TREM2 has a protective role in AD pathogenesis; its anti-inflammatory properties could reduce inflammation-induced innocent bystander neuronal damage [8, 16, 17]. In addition to the anti-inflammatory roles of TREM2, it is also known to effect phagocytosis of damaged/apoptotic cells. TREM2 interacts with endogenous ligands on neurons, leading to the direct removal of damaged cells [27]. In various models of multiple sclerosis increased microglial expression of TREM2 is associated with increased phagocytosis and a promotion of a M2-like activation state of microglia, which is thought to have protective effects [28, 29]. The removal of damaged or apoptotic neurons mediated via TREM2 could promote tissue repair in response to AD-related pathology. This TREM2 mediated phagocytic activity also has been linked to an enhanced ability of microglia to clear A*β* and amyloid plaques in vitro and in AD model APP23 Tg mice [20]. Microglia are well known to have the potential to acquire a broad array of cytotoxic and cytoprotective functional states; TREM2 appears to be important in the regulation of this balance in relation to AD pathology. 

In 2013, Jonsson et al. performed whole genome sequencing on 2261 Icelandic individuals and found that a rare mutation (rs75932628-T, frequency of 0.63%), predicted to result in a TREM2 R47H substitution, was associated with an increased risk of AD (odds ratio 2.92). Subsequently, this association was replicated in cohorts from the USA, Germany, The Netherlands, and Norway [9]. Concurrently, Guerreiro et al. confirmed the link between LOAD and the R47H variant by meta-analysis of three imputed data sets of genomewide association studies (EADI, GERAD and ANM) [7]. They also found six additional variants (Q33X, Y38C, T66M, D87D, R98W, and H157Y) that were present in affected cases and not in controls, which could be related to AD pathology. Three of these variants (Q33X, Y38C, and T66M) had been previously reported in the homozygous state to be associated with a frontotemporal dementia-like syndrome (without AD-related plaques and NFT) [30]. The critical role of TREM2 for neuronal health is highlighted by patients with autosomal recessive disorder with near complete loss of TREM2 function called polycystic lipomembranous osteodysplasia with sclerosing leukoencephalopathy (PLOSL or Nasu Hakola disease) [31–33]. Affected individuals have a progressive inflammatory neurodegenerative disorder with formation of multiple bone cysts. They typically present in the second decade of life with psychiatric symptoms and/or bone fractures, which is followed by a worsening dementia, leading to death in the 4th or 5th decades of life. The TREM2 mutation associated with this disorder (Q33X) has been identified, as discussed previously, in the heterozygous state with an increased risk of LOAD [7]. Patients with a partial loss of function of Colony-stimulating factor 1 receptor (CSF1R), which like TREM2 is a microglial receptor that signals via DAP12, have a corticobasal syndrome called hereditary diffuse leukoencephalopathy with spheroids [34]. Neither the latter disorder nor PLOSL is associated with amyloid plaques or NFT pathology; however, there is increased microgliosis along with neurodegeneration, highlighting the likely importance of the balance between microglial phagocytic and inflammatory pathways in AD.

## 3. Relationship between Tau and Trem2

Neurofibrillary tangles (NFT) are one of the pathological hallmarks of AD, which are formed by the intracellular, neuronal accumulation of abnormally aggregated and hyperphosphorulated tau protein. NFT deposition correlates better with the degree of dementia, compared to the amyloid plaque burden [35]. A number of studies have shown that increases of hyperphosphorylated tau protein (ptau) in CSF correlate with neuronal loss and is predictive of cognitive decline in AD [36–40]. A recent large GWAS study has shown that the Trem2 R47H variant has a strong association with both elevated CSF tau and ptau levels [41].

## 4. Toll-Like Receptors Structure

Toll was first identified as a receptor expressed by insects and was found to be essential for establishing dorsal-ventral orientation during embryonic development in Drosophila melanogaster [42] and for being important for defense against microbial infection [43, 44]. To date, 11 members of TLR family have been identified in humans and 13 in mice, which trigger both innate and adaptive immune responses [45–47]. Each TLR has at least one known binding ligand and/or adaptors except TLR10 [48, 49]. *TLR11*, *12*, and *1* that are not present in the human genome [50]. TLR 4 was the first mammalian homolog of Toll identified as a pattern recognition receptor required for adaptive immunity [43]. The TLRs are important for regulating microglial responses to A*β*. Fibrillar A*β* triggers microglia inflammatory cytokine production via TLR4-TLR6 heterodimers, whose assembly is regulated by CD36 [51]. Treatment of microglia with plaque material produces marked upregulation of TLR2, TLR4, TLR5, TLR7, and TLR9 mRNA [52]. 

## 5. Roles of TLR4 and TLR9

 In addition to amyloid plaques and neurofibrillary tangles that characterized AD, inflammation is observed with the progression of the disease, which is linked to production of cytokines by activated microglia. Microglia can also play a neuroprotective role by clearing A*β* via increased phagocytosis and proteolytic degradation [53–55]. Microglia can activate both innate and adaptive immune response as well express several Toll-like receptor (TLRs). TLRs play a key role in the innate immune system. Innate immunity is the first line of defense against invading microbes [56]. When a pathogen invades the body, it typically possesses a pathogen-associated molecular pattern, better known as PAMP. These PAMPs are sensed by pattern-recognition receptors (PRRs) and one specific group of PRRs is the Toll-like receptors (TLRs) [57]. TLRs are critical for eliciting an innate immune response to invading pathogens and are also important for triggering the adaptive immune responses [58, 59]. TLR engagement on antigen-presenting cells (APCs) induces cytokine release and costimulatory molecule expression that primes cells for subsequent activation and expansion of antigen-specific T cells [58–61]. TLRs also recognize a variety of danger-associated molecular patterns, called DAMPs. TLRs are expressed in all glial cells including microglia, astrocytes, oligodendrocytes, and a limited repertoire in neurons.

 TLR4 is the most actively investigated and characterized in relation to AD pathology. It recognizes microbial motifs of LPS (lipopolysaccharides) [62, 63]. The gene encoding *TLR4* in humans has 4 exons and is located on chromosome 9q32-q33, with 4 exons. TLR4 is mostly expressed in lymphocytes, monocytes, macrophages, and splenocytes [64, 65]. TLR4 has been found expressed in many other cells including epithelial [66], endothelial cells [67], and cancer cells [68, 69]. TLR4 expression on cerebral vascular endothelium increases following subarachnoid hemorrhage. TLR4 deficiency has been shown to be protective against ischemic damage and enhance neuronal survival in stroke mouse models [70]. 

 TLR9 is a protein encoded by the *TLR9* gene which has been designated as CD289. TLR9 was originally identified as a receptor that could differentially recognize bacterial DNA versus mammalian DNA, based on the high frequency of hypomethylated CpG motifs in nonmammalian DNA [71, 72]. TLR9 recognizes intracellular pathogen-derived nonmethylated CpG motifs of bacterial and viral DNA. TLR9 is expressed by several cells including dendritic cells, B lymphocytes, monocytes, and natural killer cells. TLR9 is expressed in the cytoplasm within the endoplasmic reticulum, as well as on other intracellular vesicles but not on the plasma membrane [73].

## 6. TLRs, TREM2, and Alzheimer's Disease

 Numerous studies have shown that TLRs have a crucial role in immune surveillance and inflammatory responses in the central nervous system (CNS) [12]. TLRs expression was identified on glial cells in human postmortem AD [74]. TLRs are important in AD, specifically those expressed on microglia (TLRs 1–9) [75, 76]. It has been shown that TREM2 is coupled with the immunoreceptor tyrosine-based activation motif (ITAM) sequence containing signaling adapter, DAP12, which negatively regulates TLR responses in both macrophages and dendritic cells [26, 77]. Hence, it likely TREM2 can regulate phagocytosis and/or inflammatory responses mediated via TLRs in response to AD pathology. It has been reported that TLRs on the surface of microglia cells bind A*β*, which triggers downstream intracellular signaling cascades [78, 79]. In AD patients high expression of CD14 (coreceptor for TLR4) was observed in parenchymal microglia of the frontal and occipital neocortex, hippocampus, and around senile plaques. Immunoreactivity with CD14 was detected in some perivascular areas [80]. In AD brains, high expression of TLR2 and CD14 was detected in microglia associated with amyloid plaques [74]. In AD Tg models, such as APP23, high levels of CD14 were observed in the microglia detected in the cortex and hippocampus [81]. Increased TLR4 mRNA was reported in another AD Tg model TgCRND8 [82]. In addition, TLR4-deficient mice displayed increased diffuse A*β* and fibrillar A*β* deposits compared with control mice [78], suggesting that TLR4 signaling is involved in A*β* clearance [83]. Microglia deficient in TLR2, TLR4, or the coreceptor CD14 are not activated by A*β* and do not show a phagocytic response [84]. Transgenic AD mice lacking TLR4 have markedly elevated levels of diffuse and fibrillar A*β*. Furthermore, stimulation of microglial cells with TLR2-, TLR4-, or TLR9-specific agonists accelerates A*β* clearance both in vitro and in vivo [85]. It has been reported that TLR9-mediated pathways regulate inflammatory mediator expression in astrocytes. Neurons are thought to express intracellular TLRs, including TLR9, suggesting a role for TLRs during both physiological and pathological conditions [86]. The intracerebroventricular administration of CpG in AD model Tg2576 mice has been shown to ameliorate cognitive impairments [87]. Our group has shown that the administration of the TLR9 agonist CpG oligonucleotides (ODN) containing unmethylated CpG sequences to Tg2576 mice induced a reduction of cortical and vascular A*β* levels without apparent toxicity and improved cognitive function [88]. We had previously shown that CpG stimulation is beneficial for the generation of an immune response to prion disease [89]. Several CpG DNA drugs have demonstrated good safety profiles in humans and have been tested in numerous clinical trials as antitumor, antimicrobial agents, and adjuvants in vaccines [90, 91]. Recently, we have shown that TLR9 stimulation with CpG in 3xTg AD mice with both amyloid plaque and NFT pathology greatly reduces both of these pathologies in association with cognitive benefits [92]. These results indicate that stimulation of the innate immune system through TLR9 with CpG ODN is an effective and safe method to reduce the amyloid burden and also tau-related pathology in AD model mice. 

## 7. Conclusion

 Studies conducted in the early 1990s have suggested the potentially critical role of microglia for both the formation and clearance of amyloid lesions [93–95]. Numerous studies on the relationship of TLRs to AD have shown that modification of these signaling pathways can have profound effects on AD-related pathology, through modification of the inflammatory state of microglia/macrophages. TLRs may either have a positive or negative impact on cellular responses during AD. Studies have shown that appropriate stimulation of TLR9 can ameliorate both A*β* and tau-related pathology. The recent finding by two large consortiums that a rare variant of *TREM2*, a gene which regulates phagocytosis and the activation state of microglia/macrophages, is linked to LOAD has further highlighted the important role of innate immunity in AD. This finding adds to prior data linking other genes that are associated with microglia function and a low increased risk of LOAD, such as CR1, CD33, and MS4A4A/MS4A6A [96]. These studies indicate that modification of microglial function in AD is an important therapeutic target.

## References

[1] Prince M, Bryce R, Ferri C (2011). World Alzheimer Report 2011. *Alzheimer's Disease International*.

[2] Bertram L, Tanzi RE (2012). The genetics of Alzheimer’s disease. *Progress in Molecular Biology and Translational Science*.

[3] Ridge PG, Ebbert MT, Kauwe JS (2013). Genetics of Alzheimer's disease. *BioMed Research International*.

[4] Potter H, Wisniewski T (2012). Apolipoprotein E: essential catalyst of the Alzheimer amyloid cascade. *International Journal of Alzheimer's Disease*.

[5] Verghese PB, Castellano JM, Holtzman DM (2011). Apolipoprotein E in Alzheimer’s disease and other neurological disorders. *The Lancet Neurology*.

[6] Barnes DE, Yaffe K (2011). The projected effect of risk factor reduction on Alzheimer’s disease prevalence. *The Lancet Neurology*.

[7] Guerreiro R, Wojtas A, Bras J (2013). TREM2 variants in Alzheimer's disease. *The New England Journal of Medicine*.

[8] Neumann H, Daly MJ (2013). Variant TREM2 as risk factor for Alzheimer's disease. *The New England Journal of Medicine*.

[9] Jonsson T, Stefansson H, Steinberg S (2013). Variant of TREM2 associated with the risk of Alzheimer's disease. *The New England Journal of Medicine*.

[10] Bouchon A, Hernández-Munain C, Cella M, Colonna M (2001). A DAP12-mediated pathway regulates expression of CC chemokine receptor 7 and maturation of human dendritic cells. *The Journal of Experimental Medicine*.

[11] N’Diaye E-N, Branda CS, Branda SS (2009). TREM-2 (triggering receptor expressed on myeloid cells 2) is a phagocytic receptor for bacteria. *The Journal of Cell Biology*.

[12] Arroyo DS, Soria JA, Gaviglio EA, Rodriguez-Galan MC, Iribarren P (2011). Toll-like receptors are key players in neurodegeneration. *International Immunopharmacology*.

[13] Hanke ML, Kielian T (2011). Toll-like receptors in health and disease in the brain: mechanisms and therapeutic potential. *Clinical Science*.

[14] Drouin-Ouellet J, Cicchetti F (2012). Inflammation and neurodegeneration: the story “retolled”. *Trends in Pharmacological Sciences*.

[15] Bouchon A, Dietrich J, Colonna M (2000). Cutting edge: inflammatory responses can be triggered by TREM-1, a novel receptor expressed on neutrophils and monocytes. *The Journal of Immunology*.

[16] Jiang T, Yu JT, Zhu XC, Tan L (2013). TREM2 in Alzheimer's disease. *Molecular Neurobiology*.

[17] Golde TE, Streit WJ, Chakrabarty P (2013). Alzheimer's disease risk alleles in TREM2 illuminate innate immunity in Alzheimer's disease. *Alzheimer's Research & Therapy*.

[18] Guerreiro RJ, Lohmann E, Bras JM (2013). Using exome sequencing to reveal mutations in TREM2 presenting as a frontotemporal dementia-like syndrome without bone involvement. *JAMA Neurology*.

[19] Frank S, Burbach GJ, Bonin M (2008). TREM2 is upregulated in amyloid plaque-associated microglia in aged APP23 transgenic mice. *Glia*.

[20] Melchior B, Garcia AE, Hsiung B-K (2010). Dual induction of TREM2 and tolerance-related transcript, Tmem176b, in amyloid transgenic mice: Implications for vaccine-based therapies for Alzheimer’s disease. *ASN Neuro*.

[21] Paloneva J, Manninen T, Christman G (2002). Mutations in two genes encoding different subunits of a receptor signaling complex result in an identical disease phenotype. *The American Journal of Human Genetics*.

[22] Singaraja R (2013). TREM2: a new risk factor for Alzheimer's disease. *Clinical Genetics*.

[23] Sessa G, Podini P, Mariani M (2004). Distribution and signaling of TREM2/DAP12, the receptor system mutated in human polycystic lipomembraneous osteodysplasia with sclerosing leukoencephalopathy dementia. *European Journal of Neuroscience*.

[24] Hamerman JA, Jarjoura JR, Humphrey MB, Nakamura MC, Seaman WE, Lanier LL (2006). Cutting edge: inhibition of TLR and FcR responses in macrophages by triggering receptor expressed on myeloid cells (TREM)-2 and DAP12. *The Journal of Immunology*.

[25] Turnbull IR, Gilfillan S, Cella M (2006). Cutting edge: TREM-2 attenuates macrophage activation. *The Journal of Immunology*.

[26] Hamerman JA, Tchao NK, Lowell CA, Lanier LL (2005). Enhanced toll-like receptor responses in the absence of signaling adaptor DAP12. *Nature Immunology*.

[27] Hsieh CL, Koike M, Spusta SC (2009). A role for TREM2 ligands in the phagocytosis of apoptotic neuronal cells by microglia. *Journal of Neurochemistry*.

[28] Takahashi K, Prinz M, Stagi M, Chechneva O, Neumann H (2007). TREM2-transduced myeloid precursors mediate nervous tissue debris clearance and facilitate recovery in an animal model of multiple sclerosis. *PLoS Medicine*.

[29] Piccio L, Buonsanti C, Mariani M (2007). Blockade of TREM-2 exacerbates experimental autoimmune encephalomyelitis. *European Journal of Immunology*.

[30] Guerreiro RJ, Lohmann E, Bras JM (2013). Using exome sequencing to reveal mutations in TREM2 presenting as a frontotemporal dementia-like syndrome without bone involvement. *JAMA Neurology*.

[31] Paloneva J, Kestilä M, Wu J (2000). Loss-of-function mutations in TYROBP (DAP12) result in a presenile dementia with bone cysts. *Nature Genetics*.

[32] Paloneva J, Manninen T, Christman G (2002). Mutations in two genes encoding different subunits of a receptor signaling complex result in an identical disease phenotype. *The American Journal of Human Genetics*.

[33] Fenoglio C, Galimberti D, Piccio L (2007). Absence of TREM2 polymorphisms in patients with Alzheimer’s disease and frontotemporal lobar degeneration. *Neuroscience Letters*.

[34] Rademakers R, Baker M, Nicholson AM (2012). Mutations in the colony stimulating factor 1 receptor (CSF1R) gene cause hereditary diffuse leukoencephalopathy with spheroids. *Nature Genetics*.

[35] Nelson PT, Alafuzoff I, Bigio EH (2012). Correlation of alzheimer disease neuropathologic changes with cognitive status: a review of the literature. *Journal of Neuropathology and Experimental Neurology*.

[36] Blennow K, Hampel H (2003). CSF markers for incipient Alzheimer’s disease. *The Lancet Neurology*.

[37] Hampel H, Buerger K, Zinkowski R (2004). Measurement of phosphorylated tau epitopes in the differential diagnosis of Alzheimer disease: a comparative cerebrospinal fluid study. *Archives of General Psychiatry*.

[38] De Leon MJ, Desanti S, Zinkowski R (2004). MRI and CSF studies in the early diagnosis of Alzheimer’s disease. *Journal of Internal Medicine*.

[39] Buerger K, Ewers M, Pirttilä T (2006). CSF phosphorylated tau protein correlates with neocortical neurofibrillary pathology in Alzheimer’s disease. *Brain*.

[40] Andersson C, Blennow K, Almkvist O (2008). Increasing CSF phospho-tau levels during cognitive decline and progression to dementia. *Neurobiology of Aging*.

[41] Cruchaga C, Kauwe JS, Harari O (2013). GWAS of cerebrospinal fluid tau levels identifies risk variants for Alzheimer's disease. *Neuron*.

[42] Hashimoto C, Hudson KL, Anderson KV (1988). The toll gene of drosophila, required for dorsal-ventral embryonic polarity, appears to encode a transmembrane protein. *Cell*.

[43] Medzhitov R, Preston-Hurlburt P, Janeway CA (1997). A human homologue of the Drosophila toll protein signals activation of adaptive immunity. *Nature*.

[44] Lemaitre B, Nicolas E, Michaut L, Reichhart J-M, Hoffmann JA (1996). The dorsoventral regulatory gene cassette spatzle/toll/cactus controls the potent antifungal response in Drosophila adults. *Cell*.

[45] Akira S, Takeda K (2004). Functions of toll-like receptors: lessons from KO mice. *Comptes Rendus*.

[46] Kawai T, Akira S (2006). TLR signaling. *Cell Death and Differentiation*.

[47] Kawai T, Akira S (2008). Toll-like receptor and RIG-1-like receptor signaling. *Annals of the New York Academy of Sciences*.

[48] Kopp E, Medzhitov R (2003). Recognition of microbial infection by toll-like receptors. *Current Opinion in Immunology*.

[49] Takeda K, Kaisho T, Akira S (2003). Toll-like receptors. *Annual Review of Immunology*.

[50] Kawai T, Akira S (2010). The role of pattern-recognition receptors in innate immunity: update on toll-like receptors. *Nature Immunology*.

[51] Stewart CR, Stuart LM, Wilkinson K (2010). CD36 ligands promote sterile inflammation through assembly of a toll-like receptor 4 and 6 heterodimer. *Nature Immunology*.

[52] Frank S, Copanaki E, Burbach GJ, Müller UC, Deller T (2009). Differential regulation of toll-like receptor mRNAs in amyloid plaque-associated brain tissue of aged APP23 transgenic mice. *Neuroscience Letters*.

[53] Landreth GE, Reed-Geaghan EG (2009). Toll-like receptors in Alzheimer’s disease. *Current Topics in Microbiology and Immunology*.

[54] Guillot-Sestier MV, Town T (2013). Innate immunity in Alzheimer's disease: a complex affair. *CNS & Neurological Disorders*.

[55] Wyss-Coray T (2006). Inflammation in Alzheimer disease: driving force, bystander or beneficial response?. *Nature Medicine*.

[56] Medzhitov R, Janeway C (2000). The toll receptor family and microbial recognition. *Trends in Microbiology*.

[57] Akira S (2006). TLR signaling. *Current Topics in Microbiology and Immunology*.

[58] Hoebe K, Janssen E, Beutler B (2004). The interface between innate and adaptive immunity. *Nature Immunology*.

[59] Pasare C, Medzhitov R (2003). Toll-like receptors: balancing host resistance with immune tolerance. *Current Opinion in Immunology*.

[60] Hertz CJ, Kiertscher SM, Godowski PJ (2001). Microbial lipopeptides stimulate dendritic cell maturation via Toll-like receptor 2. *The Journal of Immunology*.

[61] Boonstra A, Asselin-Paturel C, Gilliet M (2003). Flexibility of mouse classical and plasmacytoid-derived dendritic cells in directing T helper type 1 and 2 cell development: dependency on antigen dose and differential toll-like receptor ligation. *The Journal of Experimental Medicine*.

[62] Poltorak A, He X, Smirnova I (1998). Defective LPS signaling in C3H/HeJ and C57BL/10ScCr mice: mutations in *Tlr4* gene. *Science*.

[63] Kim HM, Park BS, Kim J-I (2007). Crystal structure of the TLR4-MD-2 complex with bound endotoxin antagonist eritoran. *Cell*.

[64] Opal SM, Esmon CT (2003). Bench-to-bedside review: functional relationships between coagulation and the innate immune response and their respective roles in the pathogenesis of sepsis. *Critical Care*.

[65] Aderem A, Ulevitch RJ (2000). Toll-like receptors in the induction of the innate immune response. *Nature*.

[66] Yu L, Wang L, Li M, Zhong J, Wang Z, Chen S (2010). Expression of toll-like receptor 4 is down-regulated during progression of cervical neoplasia. *Cancer Immunology, Immunotherapy*.

[67] Lagos D, Vart RJ, Gratrix F (2008). Toll-like receptor 4 mediates innate immunity to Kaposi sarcoma herpesvirus. *Cell Host and Microbe*.

[68] Kelly MG, Alvero AB, Chen R (2006). TLR-4 signaling promotes tumor growth and paclitaxel chemoresistance in ovarian cancer. *Cancer Research*.

[69] He W, Liu Q, Wang L, Chen W, Li N, Cao X (2007). TLR4 signaling promotes immune escape of human lung cancer cells by inducing immunosuppressive cytokines and apoptosis resistance. *Molecular Immunology*.

[70] Kilic U, Kilic E, Matter CM, Bassetti CL, Hermann DM (2008). TLR-4 deficiency protects against focal cerebral ischemia and axotomy-induced neurodegeneration. *Neurobiology of Disease*.

[71] Krieg AM (1995). CpG DNA: a pathogenic factor in systemic lupus erythematosus?. *Journal of Clinical Immunology*.

[72] Hemmi H, Takeuchi O, Kawai T (2000). A toll-like receptor recognizes bacterial DNA. *Nature*.

[73] Ahmad-Nejad P, Hacker H, Rutz M, Bauer S, Vabulas RM, Wagner H (2002). Bacterial CpG-DNA and lipopolysaccharides activate toll-like receptors at distinct cellular compartments. *European Journal of Immunology*.

[74] Letiembre M, Hao W, Liu Y (2007). Innate immune receptor expression in normal brain aging. *Neuroscience*.

[75] Aravalli RN, Hu S, Lokensgard JR (2007). Toll-like receptor 2 signaling is a mediator of apoptosis in herpes simplex virus-infected microglia. *Journal of Neuroinflammation*.

[76] Jack CS, Arbour N, Manusow J (2005). TLR signaling tailors innate immune responses in human microglia and astrocytes. *The Journal of Immunology*.

[77] Ito H, Hamerman JA (2012). TREM-2, triggering receptor expressed on myeloid cell-2, negatively regulates TLR responses in dendritic cells. *European Journal of Immunology*.

[78] Tahara K, Kim H-D, Jin J-J, Maxwell JA, Li L, Fukuchi K-I (2006). Role of toll-like receptor signalling in A*β* uptake and clearance. *Brain*.

[79] Cameron B, Landreth GE (2010). Inflammation, microglia, and alzheimer’s disease. *Neurobiology of Disease*.

[80] Liu Y, Walter S, Stagi M (2005). LPS receptor (CD14): a receptor for phagocytosis of Alzheimer’s amyloid peptide. *Brain*.

[81] Fassbender K, Walter S, Kühl S (2004). The LPS receptor (CD14) links innate immunity with Alzheimer’s disease. *FASEB Journal*.

[82] Walter S, Letiembre M, Liu Y (2007). Role of the toll-like receptor 4 in neuroinflammation in Alzheimer’s disease. *Cellular Physiology and Biochemistry*.

[83] Jin J-J, Kim H-D, Maxwell JA, Li L, Fukuchi K-I (2008). Toll-like receptor 4-dependent upregulation of cytokines in a transgenic mouse model of Alzheimer’s disease. *Journal of Neuroinflammation*.

[84] Reed-Geaghan EG, Savage JC, Hise AG, Landreth GE (2009). CD14 and toll-like receptors 2 and 4 are required for fibrillar A*β*-stimulated microglial activation. *Journal of Neuroscience*.

[85] Michaud JP, Halle M, Lampron A (2013). Toll-like receptor 4 stimulation with the detoxified ligand monophosphoryl lipid A improves Alzheimer's disease-related pathology. *Proceedings of the National Academy of Sciences of the United States of America*.

[86] Rolls A, Shechter R, London A (2007). Toll-like receptors modulate adult hippocampal neurogenesis. *Nature Cell Biology*.

[87] Doi Y, Mizuno T, Maki Y (2009). Microglia activated with the toll-like receptor 9 ligand CpG attenuate oligomeric amyloid *β* neurotoxicity in in vitro and in vivo models of Alzheimer’s disease. *American Journal of Pathology*.

[88] Scholtzova H, Kascsak RJ, Bates KA (2009). Induction of toll-like receptor 9 signaling as a method for ameliorating alzheimer’s disease-related pathology. *Journal of Neuroscience*.

[89] Spinner DS, Kascsak RB, LaFauci G (2007). CpG oligodeoxynucleotide-enhanced humoral immune response and production of antibodies to prion protein PrPSc in mice immunized with 139A scrapie-associated fibrils. *Journal of Leukocyte Biology*.

[90] Krieg AM (2006). Therapeutic potential of toll-like receptor 9 activation. *Nature Reviews Drug Discovery*.

[91] Bodera P, Stankiewicz W, Kocik J (2012). Synthetic immunostimulatory oligonucleotides in experimental and clinical practice. *Pharmacological Reports*.

[92] Scholtzova H, Goni F, Pan J, Sun Y, Mehta P, Wisniewski T (2012). Innate immunity stimulation as a novel therapeutic approach in Alzheimer's disease. *Alzheimer's & Dementia*.

[93] Wisniewski HM, Wegiel J, Wang KC, Lach B (1992). Ultrastructural studies of the cells forming amyloid in the cortical vessel wall in Alzheimer’s disease. *Acta Neuropathologica*.

[94] Frackowiak J, Wisniewski HM, Wegiel J, Merz GS, Iqbal K, Wang KC (1992). Ultrastructure of the microglia that phagocytose amyloid and the microglia that produce *β*-amyloid fibrils. *Acta Neuropathologica*.

[95] Wisniewski HM, Wegiel J (1994). The role of microglia in amyloid fibril formation. *Neuropathology and Applied Neurobiology*.

[96] Shi H, Belbin O, Medway C (2012). Genetic variants influencing human aging from late-onset Alzheimer’s disease (LOAD) genome-wide association studies (GWAS). *Neurobiology of Aging*.

